# Influence of Multimodal Emotional Stimulations on Brain Activity: An Electroencephalographic Study

**DOI:** 10.3390/s23104801

**Published:** 2023-05-16

**Authors:** Chenguang Gao, Hirotaka Uchitomi, Yoshihiro Miyake

**Affiliations:** Department of Computer Science, Tokyo Institute of Technology, Yokohama 226-8502, Japan; uchitomi@c.titech.ac.jp (H.U.); miyake@c.titech.ac.jp (Y.M.)

**Keywords:** brain activities, emotional stimulations, multimodal stimulations, EEG spectral and temporal analysis

## Abstract

This study aimed to reveal the influence of emotional valence and sensory modality on neural activity in response to multimodal emotional stimuli using scalp EEG. In this study, 20 healthy participants completed the emotional multimodal stimulation experiment for three stimulus modalities (audio, visual, and audio-visual), all of which are from the same video source with two emotional components (pleasure or unpleasure), and EEG data were collected using six experimental conditions and one resting state. We analyzed power spectral density (PSD) and event-related potential (ERP) components in response to multimodal emotional stimuli, for spectral and temporal analysis. PSD results showed that the single modality (audio only/visual only) emotional stimulation PSD differed from multi-modality (audio-visual) in a wide brain and band range due to the changes in modality and not from the changes in emotional degree. The most pronounced N200-to-P300 potential shifts occurred in monomodal rather than multimodal emotional stimulations. This study suggests that emotional saliency and sensory processing efficiency perform a significant role in shaping neural activity during multimodal emotional stimulation, with the sensory modality being more influential in PSD. These findings contribute to our understanding of the neural mechanisms involved in multimodal emotional stimulation.

## 1. Introduction

Multimodal emotional stimulation is widely used in modern society for evoking various emotional states in humans via external sensory inputs, including auditory, visual, olfactory, and tactile cues. These stimulations are prevalent in different media, such as movies, animation, and virtual reality, and they can be used in education, marketing, advertising, psychological counseling, and entertainment [1,2,3]. A study on the response of the human brain to these stimuli is critical for understanding the process of emotional multimodal stimulation. Electroencephalography (EEG) is commonly used to analyze and understand how emotional multimodal stimulation affects human brain activity. EEG measures the electrical activity of the brain by using electrodes placed on the scalp and recording the signals generated by neurons. By analyzing EEG data, researchers can better understand how these stimulations affect specific areas of the brain and elicit emotional responses in humans [4]. 

Brain–computer interfaces are promising for multimodal emotion recognition [5,6]. EEG signals can be used to recognize emotions from visual and audio-visual stimuli [7], and the fusion of EEG signals and audio-visual features can improve emotion recognition performance [8]. However, there is a lack of research on the process of multimodal emotional stimulation and how it affects human brain activity. Therefore, research in this area could be significant in improving the effectiveness of these stimulations. This research can assist in the development of new methods used for designing more effective emotional stimuli and for attaining a better understanding of how various multimodal stimulations impact different individuals.

Recent studies using EEG have investigated the neural correlates of emotional processing. Jiang et al. [9] found that alpha and beta oscillations in the prefrontal cortex are modulated by the valence and arousal of emotional stimuli during an affective priming task. In another study, Mu et al. [10] found that beta and gamma oscillations in the temporal and parietal regions were associated with the processing of social feedback during interpersonal trust games. Schelenz et al. [11] investigated the multisensory integration of dynamic emotional faces and voices and proposed a method for simultaneous EEG and fMRI measurements. Additionally, Li et al. [12] demonstrated that alpha oscillations in the prefrontal cortex are associated with cognitive reappraisal during an emotion regulation task, whereas Yu et al. [13] found that delta and theta oscillations in the anterior cingulate cortex and insula are modulated by the perceived intensity of emotional pain during a social exclusion task. The medial temporal pole was observed to be responsible for driving amygdala activity in response to emotional stimuli by Sonkusare et al. [14]. Ahirwal and Kose investigated correlated EEG channel agendas, and the results were examined with respect to audio-visual emotion classification [15]. In the spectral domain, Balconi and Vanutelli investigated how vocal and visual stimulation, congruence, and lateralization affect brain oscillations in emotional interspecies interactions [16]. Temporal analyses were conducted by Balconi and Carrera [17], and they showed that the cross-modal integration of an emotional face and voice leads to the P2 ERP effect. These studies highlight the continued use of EEG in elucidating the neural mechanisms underlying emotional processing. 

EEG signals have been used to investigate the neural mechanisms underlying sensory processing, including visual, auditory, and somatosensory processing. For instance, Gallotto et al. [18] investigated the neural correlates of visual perception using EEG and found that alpha and beta oscillations were modulated by attentional demands during visual processing. Craddock et al. [19] found that alpha oscillations in the somatosensory cortex were associated with the detection of tactile stimuli. Klasen et al. [20] found that emotional information processing in multimodal environments is highly integrated, with interactions and regulatory effects between different sensory channels. Additionally, Bugos et al. [21] used EEG to investigate the neural mechanisms underlying auditory processing and found that theta oscillations in the auditory cortex are modulated by musical features. Zimmer et al. [22] found that emotional cues presented in multiple sensory modalities can improve visual attention. Ross et al. [23] investigated the neural correlates of somatosensory processing using EEG and found that gamma oscillations in the somatosensory cortex are associated with the processing of vibrotactile stimulation. Brain functional area studies were utilized by Aricò et al. [24], revealing that EEG alpha power is associated with attentional changes during cognitive tasks and virtual reality immersion sensory stimulations. The effects of prolonged waking-auditory stimulation on EEG synchronization and cortical coherence were investigated by Cantero et al. [25]. Baumgartner et al. [26] found that different types of sensory stimuli evoke emotions in different ways, but the essential characteristics of emotional experience are similar. These studies demonstrate the utility of EEG in uncovering the role of oscillatory activity in sensory processing and its relationship with perception and attention.

However, previous EEG-related research has mutually and exclusively focused on the influence of stimulation modality and emotional stimuli. The effects of EEG with changes in multimodal emotional stimulations have not been thoroughly studied, especially the spatiotemporal analysis under its uni-stimulus-modality and multi-stimulus modality. Overall, much remains to be understood regarding the neural mechanisms underlying emotional and sensory processing. 

Our study aimed to investigate the changes in human brain activity in response to multimodal emotional stimulation and to assess the effects of the emotional mode and sensory modality individually and together with respect to EEG. These investigations were conducted because movies are recognized as useful naturalistic stimuli for neuroimaging studies, and they were utilized in previous studies [27]. To achieve this, we selected short film clips as the source of multimodal (visual + auditory) emotional stimulation and recruited 20 participants. We assessed two emotions (pleasure/unpleasure) and three modes (audio/visual/audio-visual); thus, a total of six conditions were assessed by EEG and self-assessment data analyses. The subjective evaluation of the self-assessment metric (SAM) and objective PSD and EEG parameters were analyzed in combination, and correlation calculations were performed to assess whether significant changes in these parameters were associated with emotional states and/or multimodality. The results of this study can be used to gain an in-depth understanding of the relevant changes in human brain activity in response to multimodal emotional stimuli, thus contributing to research and application in the areas of emotion regulation, psychotherapy, and EEG-related brain–computer interface BCI.

Highlights: This study is the first attempt to use film clips as a stimulus source for multimodal audio-visual stimulation, and it investigates the effect of three stimulation modalities on emotion regulation from the perspective of EEG. It is the first analysis of multimodal emotional stimulus EEG data in the time domain with the brain functional area and multi-band analysis and frequency domain with N200 and P300, under the uni-stimulus-modality and multi-stimulus modality of multimodal emotional stimulus, indicating specific physiological significance. 

## 2. Materials and Methods

### 2.1. Experimental Setup

#### 2.1.1. Participant Information

Referring to the previous EEG experiment using the emotion mode and sensory modality [28,29,30], twenty individuals were invited to participate in this study. Of the participants, 11 were men and 9 were women, aged 24.7 ± 1.9 years. All participants were international students at the Tokyo Institute of Technology and native Chinese speakers, right-handed, and had no history of mental illness or recent mental or physical trauma. All participants provided written informed consent to participate in this study. The study was conducted in accordance with the principles of the Declaration of Helsinki. The study was approved by the Ethics Committee of the Tokyo Institute of Technology.

#### 2.1.2. Emotional Stimulus

Next, 20 emotional video clips were selected to elicit 2 emotions: pleasure or unpleasure (sad). Each emotion was shown in 10 video clips, with each lasting 30 s. Stimulus video clips were sourced from the New Standardized Emotional Film Database for Asian Culture [31]. The detailed information for selected video clips were referred to Supplementary Material Table S1. We trimmed the original videos from the database to 30 s to meet the stimulus duration requirements for this study.

#### 2.1.3. Experimental Protocol

**Figure 1:**
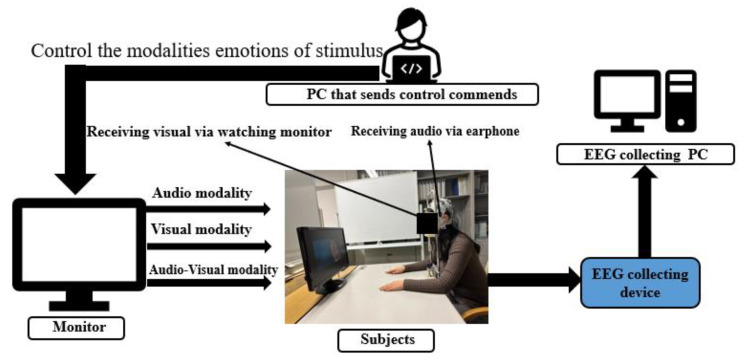
Illustration of the experimental setup.

During the experiment, subjects were asked to sit in a comfortable chair with their hands placed flat on a table. A 24-inch monitor with 1024 × 768 resolution was connected to a computer that sent control commands to play the experimental instructions and visual stimuli on the screen. The subjects were asked to wear AirPods Pro2 in the noise-canceling mode while playing audio stimuli. The distance between the participants and the monitor was approximately 90 cm, measured with their chins resting on a chinrest to prevent the shaking of the head. The experimental setup is shown in Figure 1.

Before the experiment, a questionnaire survey was conducted to ascertain the physical and mental condition of the subjects. The contents of questionnaire were referred to supplementary material: S3. Questionnaire before emotion EEG experiment. After the questionnaire, according to the type of stimulus, there were three experimental conditions/modalities: audio stimulus, visual stimulus, and audio-visual stimulus. Resting conditions were used as the control.

Audio stimulus modality: The film clip was played with a black screen, and only audio was provided.

Visual stimulus modality: The film clip was played while muted, and only visual stimulus was provided.

Audio-visual stimulus modality: The film clip was played with original audio and visual information.

Resting state: No audio or visual information was provided.

Each subject first performed the experiment in the resting state to enable the measurement of EEG in the resting state. In the resting state, the subjects maintained a sitting posture that was the same as that in stimulus-corresponding tasks. The duration for the resting state EEG recording was 300 s.

**Figure 2:**
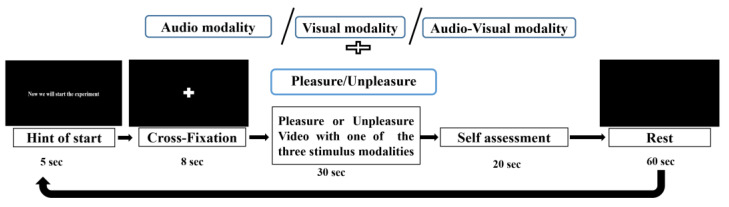
Workflow for stimulation representation.

The experimental workflow for the three stimulus modalities of multimodal emotional stimulation is shown in Figure 2.

The experimental execution of the multimodal emotional conditioning task consisted of 60 trials, each lasting approximately 2.5 min. At the beginning of each trial, a 5 s cue indicated the starting time, followed by an 8 s cross-fixation to direct the participant’s attention. The stimulus lasted 30 s and was pseudo-randomly presented with one of two emotional stimuli (pleasure or unpleasure) in one of the three stimulus modalities. Pseudo-random protocol was referred to Supplementary Material: S2. Pseudo-random protocol for the experiment. After the presentation of the stimulus, participants completed a self-assessment questionnaire to rate the type and degree of emotion experienced, spending approximately 20 s. Then, there was a 60 s rest without any visual or auditory stimulation. For each subject, the entire experiment, which included the requisite rest during the emotional conditioning task and resting state EEG measurements, lasted approximately 2.5 h; a 5 min break was provided after every 20 trials to avoid fatigue effects.

### 2.2. Data Acquisition 

#### 2.2.1. EEG Data Acquisition

**Figure 3:**
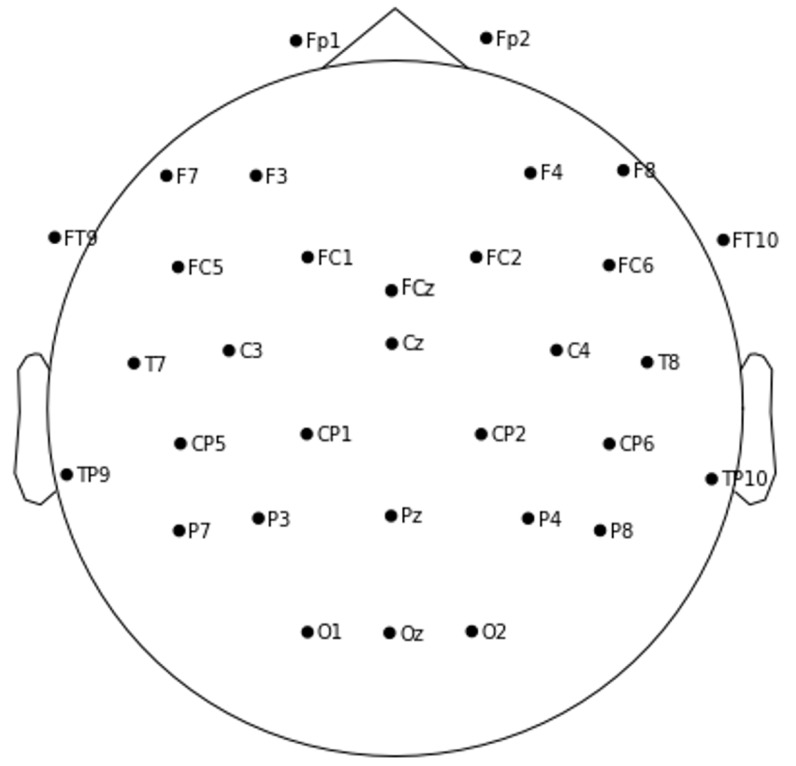
EEG electrode distribution.

In this study, a 32-channel EEG system (Brain Products GmbH, Gilching, Germany) was used to collect EEG data, and the electrodes were as follows: Fp1, Fp2, F3, F4, F7, F8, FT9, FT10, FC5, FC6, T7, T8, CP5, CP6, TP9, TP10, P7, P8, FC1, FCz, FC2, C3, Cz, C4, CP1, CP2, P3, Pz, P4, O1, O2, and Oz. The positions of the channels on the scalp are shown in Figure 3.

Before the experiment, each subject wore a headset for EEG collection. By the application of a special gel to the scalp, the impedance between each electrode and the scalp was reduced to below 10 kΩ. Using BrainVision Recorder software, an amplifier was used to amplify weak electrical signals from the brain, and the data acquisition system was used to digitize and store the data. We used FCz as the reference electrode, with a sampling frequency of 500 Hz. The data from these experiments are detailed in Table 1.

EEG data were divided into seven categories. Audio unpleasure EEG data were collected with the audio stimulus modality, and film clips were collected with unpleasure emotions. For visual unpleasure data, EEG data were collected with the visual stimulus modality, and film clips were collected with unpleasure emotions. For audio-visual unpleasure data, EEG data were collected with audio-visual stimulus modalities, and film clips were collected with unpleasure emotions. For audio pleasure data, EEG data were collected with the audio stimulus modality, and film clips were collected with pleasure emotions. For visual pleasure, EEG data were collected with the visual stimulus modality, and film clips were collected with pleasure emotions. For audio-visual pleasure, EEG data were collected with the audio-visual stimulus modality, and film clips were collected with pleasure emotions. Resting state EEG data were collected in the resting state.

Collected data comprised the following: 20 (subjects) × 2 (emotional classes) × 3 (stimulus modalities) × 10 (trials) = 1200 trials, with 200 trials for each emotion with 1 stimulus modality. The duration of each trial was 30 s. Resting state = 1 × 20 (subjects) trials were collected before the main experiment with a duration of 300 s for each trial. 

#### 2.2.2. Self-Assessment Data 

**Figure 4:**
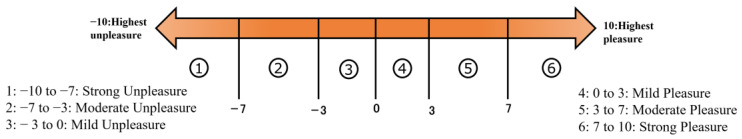
Self-assessment metric.

In each trial of the multimodal emotional conditioning tasks, self-assessment data were collected post-stimulus. According to the self-assessment metric (SAM) [32], the participants’ self-reported scores during each trial, as shown in Figure 4. 

During the self-assessment in each trial, subjects named the level of emotional arousal they experienced and assigned an integer score from 0 to 10. If the participant observed that the emotion was pleasure, it was recorded as the original score, and if the emotion was unpleasure, it was recorded as a negative absolute value of the score. All readings were recorded by the researcher.

Self-assessment data were used as a subjective index to validate multimodal emotional stimulation, and the data were explored using EEG analysis.

### 2.3. Data Analysis 

#### 2.3.1. EEG Data Preprocessing

When preprocessing EEG data to enhance data quality, we utilized several methods to improve the accuracy and reliability of the data. Specifically, the following steps were taken:

Filtering: We applied a notch filter with a frequency of 50 Hz to remove electromagnetic interference from the EEG data. Additionally, we used the average potential as the reference electrode and re-referenced the EEG electrodes to further enhance data quality. These steps helped reduce noise and artifacts in the EEG signals.

Artifact removal: We identified and removed EEG signals that were greater than 100 µv, which are commonly considered noise in EEG data. This step helped further reduce the impact of artifacts on the data.

ICA decomposition: We used independent component analysis (ICA) to identify and separate the different independent sources of the EEG signals. This step helps identify components that are not related to the neural activity of interest, such as eye movements or muscle artifacts. The ICA result would be used in ICLabel. 

ICLabel: We used the ICLabel tool in EEGLAB [33] to classify the independent components obtained by ICA. ICLabel is a commonly used automatic classification tool that classifies independent components into different categories, including eye movement, muscle artifact, and neural activity. We used ICLabel to remove components corresponding to eye movements and muscle artifacts in order to improve the accuracy of our results.

By utilizing these methods, we were able to preprocess the EEG data and prepare it for further analysis. The combination of filtering, artifact removal, ICA decomposition, and ICLabel classification helped enhance data quality and reduce the impact of noise and artifacts on the data.

#### 2.3.2. Power Spectral Density (PSD) Analysis of EEG

For the analysis of EEG data after preprocessing, we first conducted frequency domain analysis.

In frequency domain analysis, the main index used was PSD, which is the result of the spectral analysis of the power of EEG signals. Different frequency bands of EEG can be obtained using the above experimental conditions and the distribution of PSD in different regions; thus, the significance of brain activity under different experimental conditions can be specifically explained [34]. Meanwhile, the inner condition comparison can be easily conducted using PSD. Before explaining the PSD calculation method adopted in this study, we specify the strategy for the PSD analysis. As this experiment pertained to emotion and stimulation modalities, we used frequency band and brain functional analyses together. We aimed to reveal the conditions under different frequency ranges and the activity of each functional brain area corresponding to the EEG obtained from different experimental conditions.

##### EEG Spectral Analysis

EEG spectrum band research is a well-recognized analysis method in frequency domain studies and has significance in the study of emotion and sensation. In this EEG, signals are divided into various frequency bands [35]. 

Commonly used sub-spectral bands include delta: 1–4 Hz; theta: 4–8 Hz; alpha: 8–14 Hz; beta: 14–30 Hz; and gamma: 30–100 Hz [36]. In this EEG study with emotional and multimodal (visual, auditory, and audio-visual) stimulations, we selected three bands—delta, theta, and alpha—and used a bandpass filter to obtain sub-spectrum bands. 

Increases in delta and theta waves are associated with negative emotions, such as unpleasure, while increases in alpha waves are associated with positive emotions, such as pleasure [37]. In addition, different stimulation modalities can elicit different EEG patterns that can be used to differentiate stimulus types, such as visual, auditory, or multimodal stimuli. These frequency bands are particularly relevant in the analysis of emotion and stimulus patterns as they provide valuable insights into the physiological changes that occur in response to different emotional and stimulus conditions [38]. By analyzing these specific frequency bands, we can better understand the neural mechanisms underlying emotional processing, such as unpleasure and pleasure.

For example, we can compare pleasure and unpleasure emotional stimuli by comparing the intensity changes in different frequency bands. In addition, we compared the effects of visual, auditory, and audio-visual stimuli on different frequency bands, thereby assessing the effects of different stimulation modalities on emotional and sensory responses.

##### EEG Functional Brain Mapping 

**Figure 5:**
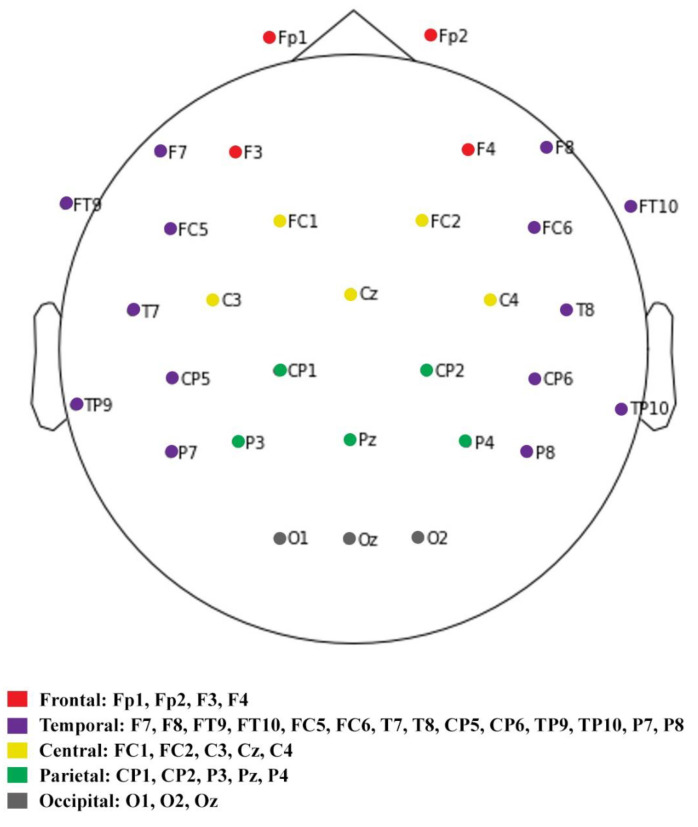
Brain functional area allocation with EEG electrodes.

The study of divided functional brain areas is important in the field of brain science [39]. Functional brain division refers to the subdivision of the entire brain into smaller regions as per their contribution to different functions. In this study, five functional areas were defined as corresponding to the EEG channels utilized in the experiment: prefrontal, temporal, central, parietal, and occipital [40]. With the exception of the original reference channel FCz, the remaining 31 channels were allocated as per Figure 5.

The prefrontal cortex (Fp1, Fp2, F3, and F4) is mainly responsible for thought, cognition, and behavioral control.

The temporal cortex (F7, F8, FT9, FT10, FC5, FC6, T7, T8, CP5, CP6, TP9, TP10, P7, and P8) is primarily responsible for language processing, auditory cognition, and memory processing.

The central cortex (FC1, FC2, C3, Cz, and C4) is primarily responsible for functions, such as perception, sensation, and sensory fusion.

The parietal cortex (CP1, CP2, P3, Pz, and P4) is primarily responsible for body perception, spatial orientation, and hand–eye coordination.

The occipital cortex (O1, O2, and Oz) is primarily responsible for visual processing and cognition.

For each trial, the processes of a one-channel EEG with bandpass filtering and its PSD were calculated using the Welch method. The following process was performed using the MNE library in Python [41,42].

Segmentation: We divided the pre-processed EEG signals using the default setting for calculating the PSD using Welch’s method in a library, which usually includes overlapping windows. Adjacent windows overlapped by a certain percentage of their length, typically 50%. This allowed for a more accurate estimation of the PSD by reducing variance.

PSD estimation: We used a function for estimating PSD using Welch’s method to estimate the PSD of each window of each EEG channel. The default settings for this function included overlapping windows, a Hanning window, and a Welch window size of 250.

Denoting a one-channel EEG from one trial as vector *X*, the PSD can be determined as follows:

(1) $$p_{psd\left(\omega \right)=\frac{1}{n}|{\sum_{m=0}^{n-1}X\left(m\right)\cdot e^{-jwm}|}^{2}}$$

where *X* is a vector with n elements, as indicated by the recordings from one channel. 

**Figure 6:**
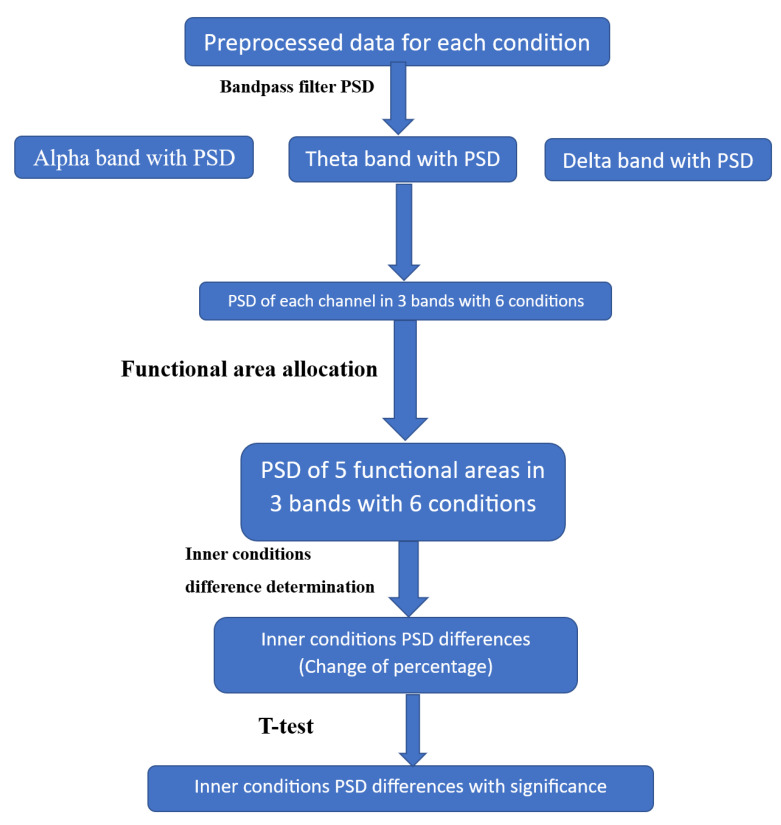
Block diagram of spectral EEG analysis.

Average PSD: The function for estimating the PSD using Welch’s method returns an average PSD estimate for each channel in each window. The average PSD was calculated from the PSD estimates across all windows for each channel. Using the process shown in Figure 6, we obtained the PSD for each channel in each frequency band under different experimental conditions.

#### 2.3.3. Event-Related Potential Analysis for Temporal EEG 

For this study, we selected six channels (Cz, C3, C4, P3, Pz, and P4) for the analysis of event-related potentials (ERPs). ERPs are time-locked electrical responses of the brain that occur in response to specific events, such as the presentation of a visual or auditory stimulus. The N200 and P300 ERP components are well-established indicators of cognitive processing [43].

The N200 component occurs approximately 200 ms after a stimulus and is typically associated with early sensory processing. It reflects the brain’s response to the physical features of a stimulus, such as brightness or pitch. In contrast, P300 occurs approximately 300 ms after the stimulus and reflects the allocation of attention and semantic processing. It is often used to measure cognitive processing related to decision making, working memory, and attention [44].

**Figure 7:**
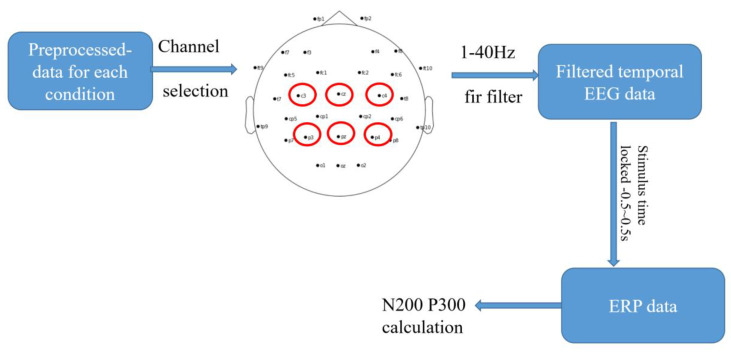
Block diagram of temporal EEG analysis.

In this study, we focused on N200 and P300 components and selected the six commonly used channels for analyses. The channels selected were Cz, C3, C4, P3, Pz, and P4. We aimed to investigate the differences in ERP responses between conditions and determined whether there were any significant differences in cognitive processing [45]. The process for temporal EEG analyses is shown in Figure 7.

## 3. Results and Discussion

### 3.1. Self-Assessment 

First, considering the subjective self-assessment of emotion induced by multimodal emotional simulation, in 20 subjects, each person experienced 60 trials of stimulation; the total number of stimulation conditions was 6, 2 (pleasure/unpleasure) × 3 (audio/visual/audio-visual). The self-assessment results for each subject in terms of the experimental conditions are shown in Table 2.

By calculating the self-assessments of all subjects for the six experimental conditions, we obtained the average self-assessment score [46,47] for each experimental condition shown in Table 2.

We refer to pleasure as PL and unpleasure as UNPL. The results of the self-assessment of all subjects in the six conditions are audio pleasure = 6.50 ± 2.40; visual pleasure = 6.17 ± 2.51; audio-visual pleasure = 7.23 ± 1.91; audio unpleasure = −6.17 ± 2.35; visual unpleasure = −5.91 ± 2.30; and audio-visual unpleasure = −7.00 ± 2.02, repsecitvely. By using one-way ANOVA [48], we first validated the significance of the self-evaluation of the six conditions, where the basic elements of each condition were each subject; that is, we used 20 elements for each set of data, with a total of 6 sets of data. The obtained p-value was 9.43 × 10^−78^ < 0.1, which shows that, in the self-assessment results of the six conditions, there are significant differences within the 20 subject groups. 

Next, we calculated the relative difference between the two pairs of conditions within the six conditions. The *p*-values from the one-way ANOVA were as follows: for audio pleasure and visual pleasure, *p* = 0.48 > 0.1, indicating no significant difference; for audio unpleasure and visual unpleasure, *p* = 0.61 > 0.1, indicating no significant difference; for audio pleasure and audio-visual pleasure, *p* = 0.06 < 0.1, indicating a significant difference; for visual pleasure and audio-visual pleasure, *p* = 0.02 < 0.1, indicating a significant difference; for audio unpleasure and audio-visualu npleasure, *p* = 0.01 < 0.1, indicating a significant difference; and for visual pleasure and audio-visual pleasure, *p* = 0.02 < 0.1, indicating a significant difference.

According to the results of one-way ANOVA, we concluded that the audio and visual conditions have no significant difference (*p* > 0.1) for the two emotional states of pleasure and unpleasure. However, the audio-visual condition was significantly different (*p* < 0.1) from audio- and visual-only conditions.

The results also suggest that a combination of multiple sensory modalities (audio-visual modality) can have a greater impact on mental states than stimulation from a single sensory modality (audio only or visual only) in view of subjective assessments.

### 3.2. Comparison of PSD between Resting EEG and Multimodal Stimulation EEG 

**Figure 8:**
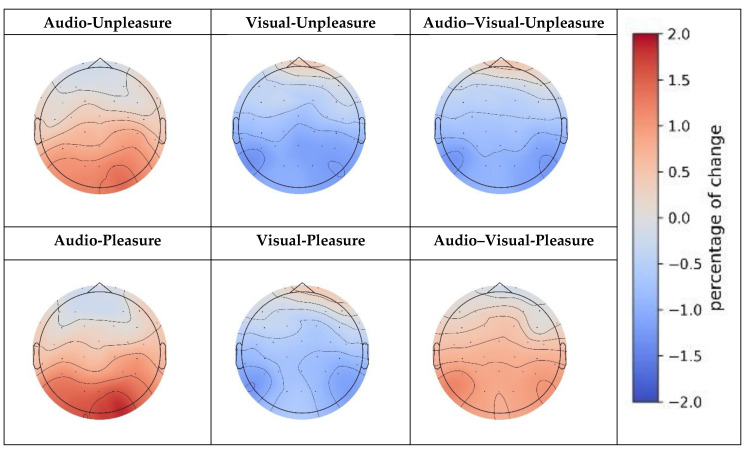
Topography distribution for change in PSD with pre-stimulated/stimulated conditions.

To explore the neurological effects of multimodal emotional stimulation in the six conditions, we first studied the PSD during stimulated and pre-stimulated phases. We chose the start of the stimulation as 0 s, an EEG of 0~30 s for 3000 ms EEG in the stimulated phase, and the 500 ms EEG of −5~0 s for the pre-stimulated phase. The stimulus EEG and pre-stimulus EEG were transformed by epoching original EEG data. The changes before and after stimulation were compared. The extracted stimulus and pre-stimulus EEG datasets were preprocessed, and the PSD of the band-divided brain functional area was calculated to obtain the pre-stimulated and stimulated PSD in decibels. We calculated the percentage change in PSD. For each frequency band interval corresponding to each experimental stimulus condition and functional area, we used the following formula:

(2) $${Change\;of\;percentage}_{PSD}=\frac{{stimulated}_{PSD}-{prestimulated}_{PSD}}{\left|{prestimulated}_{PSD}\right|}\times 100\%$$

where the ${prestimulated}_{PSD}$ had a negative value, and we used its absolute value for correction. For the data of 20 subjects, we calculated their respective results and used a paired *t*-test to assess statistical significance. We considered results with a *p*-value < 0.1 as significantly different and denoted 0.05 < *p* < 0.1 as *, 0.01 < *p* < 0.05 as **, and 0.01 > *p* as ***. The results are shown in Table 3 and Figure 8.

There was a statistically significant difference between the stimulated and pre-stimulated PSD only in the frequency domain of the alpha wave and none in those of the delta and theta bands. Therefore, we chose only the alpha frequency domain and drew the topography of ${Change\;of\;percentage}_{PSD}$. The six conditions were as follows.

The occipital PSD increased by 2.177 in the presence of both auditory stimulation and unpleasure. Temporal, parietal, and occipital PSDs decreased by 1.814, 2.449, and 2.845, respectively, under visual stimulation and unpleasure moods. The PSD in the temporal, parietal, and occipital lobes decreased by 1.514, 1.867, and 2.176, respectively, under audio-visual stimulation and unpleasure moods. 

Temporal and occipital PSDs increased by 1.465 and 2.696, respectively, under auditory stimulation and pleasure. Temporal, parietal, and occipital PSDs decreased by 1.347, 1.759, and 1.876, respectively, under visual stimulation and pleasure. Under audio-visual stimuli and pleasure emotions, parietal, parietal, and occipital PSDs increased by 0.359, 0.473, and 0.07, respectively.

These results suggest that changes in the PSD in the brain are influenced by sensory and emotional factors and that the effects of these factors vary by brain region and specific sensory and emotional conditions.

In the pre-stimulated and stimulated conditions for comparison, only the alpha wave showed a statistically significant difference among the selected bands. This indicates that different frequency bands in the EEG are related to different brain processes. Alpha is associated with attention and relaxation, and changes in alpha activity are associated with different cognitive and emotional states [49]. Thus, PSD changes in the alpha band may be more sensitive to sensory and emotional conditions than those in the other bands, leading to the observations. The results show that changes in the PSD of the brain under different sensory and emotional conditions are complex and multifaceted.

In terms of sensory conditions, the results suggest that auditory stimuli have different effects on the brain than visual stimuli. For example, PSD in the temporal and occipital regions increased in response to auditory stimulation, whereas PSD in the temporal, parietal, and occipital regions decreased in response to visual stimulation. In response to audio-visual stimuli, the PSD increased in the temporal regions but decreased in the parietal and occipital regions. These results suggest that different sensory inputs have different effects on brain function and that the combination of auditory and visual stimuli can produce unique patterns of PSD changes.

In terms of emotional conditions, the results suggest that unpleasure and pleasure emotions affect the brain differently. Under the unpleasure condition, the PSD was reduced, with the greatest reduction in the occipital regions. In contrast, in the pleasure condition, the PSD increased, with the greatest increase in the occipital region. These results suggest that emotions have profound effects on brain function and that different emotions can produce different patterns of PSD changes.

### 3.3. Emotion-Related PSD Analysis

To explore the role of the emotional part of the stimulus in this experiment, we performed emotion-related PSD analyses. We divided the control group into pleasure and unpleasure for each modality stimulation so that we could obtain a total of three pairs of comparisons: Audio Pleasure vs. Audio Unpleasure, Visual Pleasure vs. Visual Unpleasure, and Audio-Visual Pleasure vs. Audio-Visual Unpleasure. We chose the start of stimulation as 0 s and an EEG of 0~30 s for 3000 ms in the stimulated phase for both pleasure and unpleasure conditions. The PSD of each functional area was calculated for the three theta, beta, and alpha bands. The percentage change was used to measure the change in the pleasure state compared to the unpleasure state. The calculation was as follows:

(3) $${Change\;of\;percentage}_{PSD}=\frac{{Pleasurestimulated}_{PSD}-{Unpleasurestimulated}_{PSD}}{\left|{Unpleasurestimulated}_{PSD}\right|}\times 100\%$$

Since the calculated ${Unpleasurestimulated}_{PSD}$ had a negative value, we used its absolute value to ensure the correction of results. We calculated the respective results for the data from the 20 subjects and used the t-test for statistical significance. Results with a *p*-value < 0.1 were considered statistically significant, and we denoted 0.05 < *p* < 0.1 as *, 0.01< *p* < 0.05 as **, and 0.01 > *p* as ***. The calculated results are shown in Table 4.

**Figure 9:**
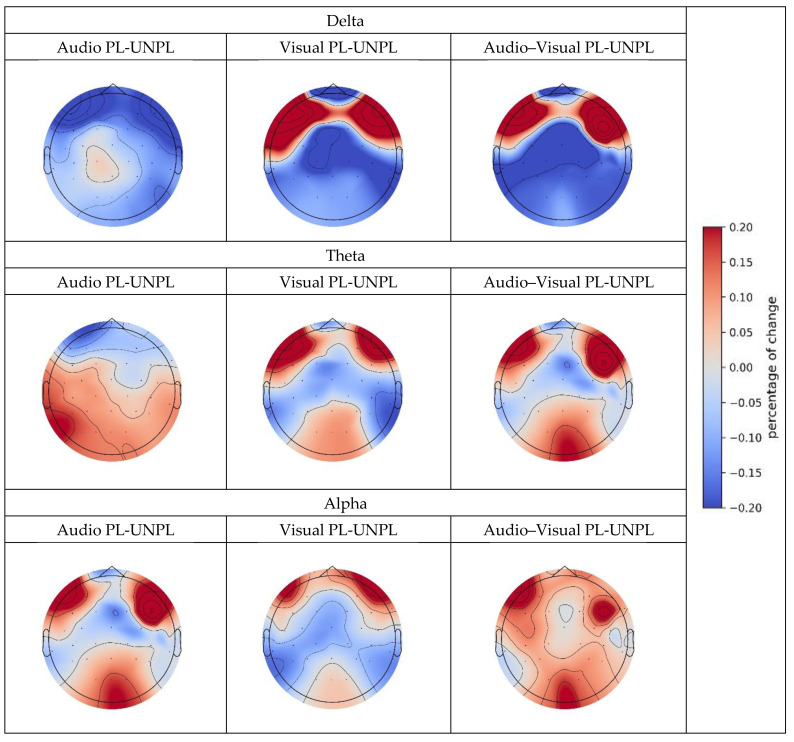
Topography distribution for changes in PSD with different emotional conditions.

As there were statistically significant differences in the range of the three bands, we drew the topography of the percentage change in the three bands, as shown in Figure 9.

We observed that the results of the delta interval were significantly different from those of theta and alpha. We concluded that in the delta band and parietal and occipital brain regions, the pleasure PSD was greater than the unpleasure PSD. However, in the theta and alpha bands and the parietal and occipital regions, pleasure PSD was lower than unpleasure PSD. The influence of each sensory stimulation modality differed among the three bands. The difference between theta with alpha patterns is evident from the topography diagram. Although the changing trend of theta is approximately the same as that of alpha, the degree of change in alpha is greater than that of theta; that is, alpha is more sensitive to changes in emotions.

Combining the results of the t-test and functional brain area calculations, we can observe the following.

Delta band: The PSD of the delta wave changed significantly under the audio and audio-visual modalities; however, no similar change was observed under the visual modality. In the audio modality, the PSD of delta waves did not change significantly in the frontal and central regions but was slightly reduced by 0.171% in other regions. These results suggest that delta waves may be involved in emotional processing in certain contexts.

Theta band: The PSD of the theta waves changed significantly in all audio, visual, and audio-visual modalities. In the audio modality, the PSD increased in the temporal, central, and parietal regions (0.148%, 0.174%, and 0.221%, respectively) and decreased in the occipital region (−0.234%). Under visual conditions, the PSD increased in the temporal and parietal regions (0.134% and 0.238%, respectively) but did not change significantly in other regions. In the audio-visual modality, the PSD increased in the central and temporal regions (0.123% and 0.104%, respectively). These results suggest that theta waves are sensitive to emotional states.

Alpha band: The PSD of the alpha band changed significantly in all audio, visual, and audio-visual modalities. In the audio modality, the PSD increased in the central and parietal regions (0.362% and 0.699%, respectively) but did not change significantly in the temporal regions. In the visual mode, the PSD increased (0.269%) in the occipital region and decreased (−0.104%) in the temporal region, with no significant changes in other regions. In the audio-visual condition, the PSD increased in the central and occipital regions (0.0492% and 0.034%, respectively) and decreased in the temporal and parietal regions (−0.031% and −0.130%, respectively). 

The results indicated that the change in emotional state had a significant impact on the change in theta activity. The PSD in the central and parietal regions significantly increased under the unpleasure condition, while the theta activity in the temporal and parietal regions decreased under the pleasure condition. This suggests that changes in the theta activity, a hallmark of emotional processing in the brain, may be more complex than previously thought. The specific regions affected by changes in theta activity may vary according to the emotional state.

Furthermore, the results indicated that the change in emotional state also had a significant impact on the change in alpha activity. The PSD of the occipital region significantly increased with respect to the pleasure emotion, whereas the alpha activity of the temporal region significantly decreased with respect to the unpleasure emotion. These results suggest that alpha activities may be involved in emotional processing, particularly in the occipital region, which is thought to be involved in visual processing. The specific regions affected by changes in alpha activity may depend on emotional state and sensory input [50].

Findings in the delta band suggest that changes in the emotional state may be less sensitive to changes in the delta activity than in other bands. The PSD in the frontal regions was significantly lower in the pleasure emotion, suggesting that changes in delta activity may be related to emotional processing within this brain region [51]. The specific regions affected by changes in delta activity may vary according to the emotional state and sensory input.

Thus, the effect of emotion on PSD could be discussed in that the analysis found that the emotional state of the participants had a significant impact on PSD, with all three frequency bands (delta, theta, and alpha) showing changes in activity that were dependent on the emotional state. Emotion perception shows serial dependence within and between sensory modalities [52]. This suggests that emotions can modulate neural activity in specific brain regions and frequency bands, which may have implications for understanding emotional processing and regulation.

This section investigated the changes in PSD in response to emotional stimuli across different frequency bands, focusing on the emotional states of pleasure and unpleasure. We explored the changes in PSD across different sensory inputs and functional brain regions to better understand the complex interplay between emotional states and brain functions. The findings suggest that different frequency bands exhibit different patterns of change in response to emotional stimuli and that the specific affected regions also vary relative to the emotional state and sensory input. 

### 3.4. Stimulus-Modality-Related PSD Analysis

Next, we focused on the influence of the stimulus modality on PSD in the frequency domain. We used multimodality stimuli (audio-visual) and single-modality stimuli (audio and visual) in our experiment. That is, we wanted to compare the changes in PSD between the multimodality stimulus and the two single-modality stimuli. Therefore, we performed comparisons in four categories: audio-visual unpleasure vs. audio unpleasure (**AV-A-UNPL**), audio-visual pleasure vs. audio pleasure **(AV-A-PL**), audio-visual unpleasure vs. visual unpleasure (**AV-V-UNPL**), and audio-visual pleasure vs. visual pleasure (**AV-V-PL**). Here, we used the percentage change to evaluate the change in PSD as well; that is, we evaluated the change in PSD after adding additional stimuli (multimodal) compared with a single modality, which is calculated as follows.

(4) $${Change\;of\;percentage}_{PSD}=\frac{{multistimulated}_{PSD}-{Unistimulated}_{PSD}}{\left|{Unistimulated}_{PSD}\right|}\times 100\%$$

Since the calculated ${unitimulated}_{PSD}$ had a negative value, we used its absolute value to ensure the correction of results. For the data of 20 subjects, we calculated their respective results and used the t-test to assess statistical significance. Results with a *p*-value < 0.1 were considered statistically significant, and we denoted 0.05 < *p* < 0.1 as *, 0.01< *p* < 0.05 as **, and 0.01 > *p* as ***. The calculated results are shown in Table 5.

**Figure 10:**
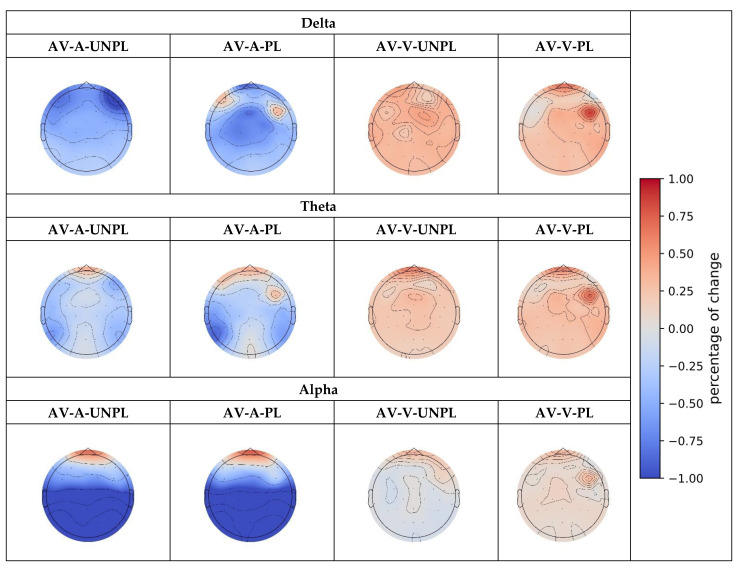
Topography distribution for the change in PSD with multi-stimulated/uni-stimulated pleasure/unpleasure conditions.

As there were statistically significant differences in the range of the three bands, we drew the topography of the percentage change in the three bands, as shown in Figure 10.

**Figure 11:**
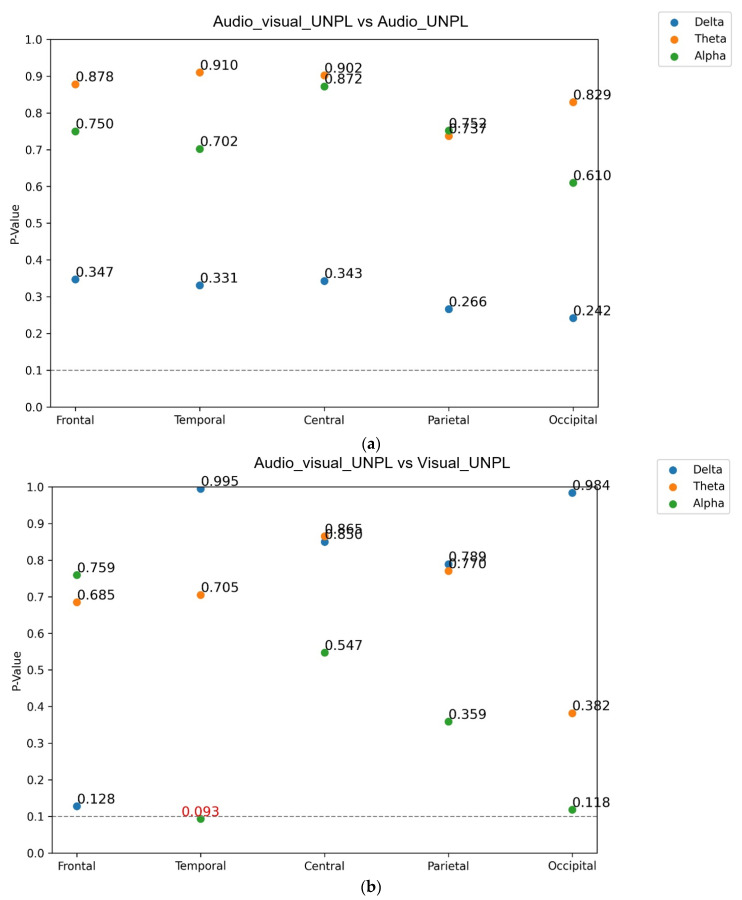

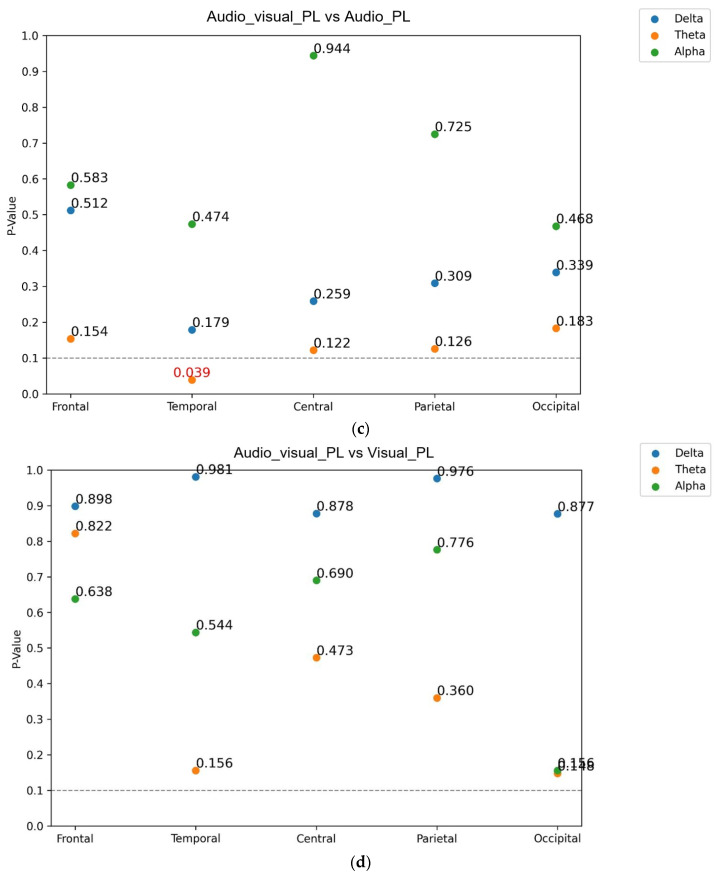
*p*-value from the Pearson correlation analysis between SAM and PSD in the multi-stimulated/uni-stimulated pleasure/unpleasure conditions. The four comparative pairs are represented in each sub-figure (**a**–**d**), and each figure consists of three frequency bands and five brain functional area results.

Although the apparent difference is the additional stimulus in the four comparisons, there are also some differences between single-modal and multimodal emotions. We aimed to understand whether these differences in emotions were the cause of significant differences in PSD. Therefore, we used Pearson correlation analysis to analyze whether the change in emotion was influenced by the PSD that is attributed to single or multimodal stimulation. We used the Pearson correlation method and *t*-test (one-way ANOVA) to calculate the *p*-value and evaluated correlations in all cases [53]. The results are represented in Figure 11.

The SAM-PSD correlation analysis revealed that the addition of visual to audio stimuli increased brain activity in the frontal and parietal regions, whereas the addition of audio to visual stimuli increased brain activity in the temporal and occipital regions. These findings suggest that the saliency of the emotional cue and the processing efficiency of the sensory modality perform important roles in shaping neural activity patterns. Moreover, the Pearson correlation analysis revealed that changes in emotions were not the direct cause of the significant differences in PSD, indicating that the differences in PSD are likely due to differences in the stimuli themselves.

From these results, we concluded that only correlation results showed statistically significant differences and that these two results did not show significant differences in the PSD *t*-test. Therefore, we concluded that, in the comparison of stimulation modalities, the difference in PSD comes from the difference in the stimulus itself and does not directly come from a change in emotion. Therefore, we combined the previous results to arrive at the following analysis.

Delta (1–3 Hz):

In the unpleasure state, adding visual to audio stimuli led to a statistically significant decrease in delta activity across all brain regions ranging from 0.8126% to 1.2049%, respectively. Adding audio to visual stimuli led to a statistically significant increase in use activity in the central, parietal, and occipital regions, ranging from 0.5585% to 0.6931%, respectively. In the pleasure state, adding visual to audio stimuli led to a statistically significant decrease in activity in the central, parietal, and occipital regions ranging from 1.3092% to 1.5284%. Adding audio to visual stimuli led to a statistically significant increase in activity in the frontal, temporal, and occipital regions ranging from 0.3061% to 0.5248%, respectively.

Theta (4–7 Hz):

In the unpleasure state, adding visual to audio stimuli led to a statistically significant increase in theta activity in the frontal and parietal regions, ranging from 0.1514% to 0.7576%, respectively, with a statistically significant *p*-value of less than 0.05 in the occipital region. Adding audio to visual stimuli led to a statistically significant increase in activity in all regions ranging from 0.2996% to 0.8381%, respectively. In the pleasure state, adding visual to audio stimuli led to a statistically significant increase in activity in the frontal and parietal regions ranging from 0.2488% to 0.7675%, respectively. Adding audio to visual stimuli led to a statistically significant increase in activity in the temporal and occipital regions ranging from 0.3063% to 0.4241%, respectively.

Alpha (8–13 Hz):

In the unpleasure state, adding visual to audio stimuli led to a statistically significant increase in alpha activity in the frontal, parietal, and occipital regions ranging from 0.3093% to 4.4406%, respectively. Adding audio to visual stimuli led to a statistically significant increase in activity in all regions ranging from 0.1413% to 0.2344%, respectively. In the pleasure state, adding visual to audio stimuli led to a statistically significant increase in activity in the frontal and parietal regions ranging from 0.2535% to 5.0590%, respecively. Adding audio to visual stimuli led to a statistically significant increase in activity in the temporal and occipital regions ranging from 0.0259% to 0.4241%, respectively.

Therefore, the three bands showed significant changes in activity when audio and visual stimuli were combined compared to when they were presented separately. In addition, all three bands showed changes in activity that were dependent on the emotional state of participants.

**Figure 12:**
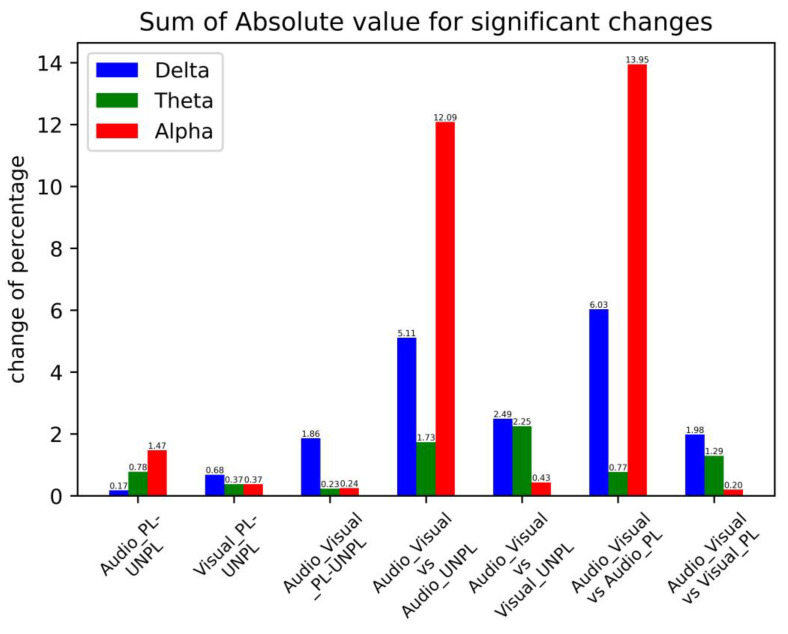
Sum of the absolute value for significant changes corresponding to emotional change pairs and stimulus modality change pairs. The three on the left are emotional changes, and the four on the right are stimulus modality changes.

However, some differences were observed between bands. Delta activity consistently decreased when visual stimuli were added to audio stimuli across all regions and emotional states. Theta activity significantly increased in the frontal and parietal regions when audio stimuli were combined with visual stimuli, regardless of the emotional state. The alpha activity showed more complex changes, with different regions and emotional states showing different patterns. In summary, these results suggest that the addition of different sensory inputs can modulate neural activity in specific brain regions. The pattern of changes in neural activity in response to different sensory inputs varies across the different frequency bands and brain regions. Overall, we compared three pairs for emotional changes and four pairs for stimulus modality changes. We determined all statistically significant changes in percentages in the PSDs. The results corresponding to each band for each condition are summarized in Figure 12.

The above figure shows that the PSD of the three groups on the left and the emotion-related changes are calculated and compared with the PSD of the four groups on the right, where changes are related to the stimulus modality. For the significant changes in the PSD with regard to stimulus modality (i.e., audio or visual), the change due to the stimulus modality was more significant than that due to the emotional state. It can be concluded that in multimodal emotional stimulations, the effect of change in the stimulation modality itself is significantly greater than the change in emotion. 

Therefore, the effect of stimulus modality on PSD could be discussed as follows: The analysis also found that the addition of different sensory inputs (audio or visual) can modulate neural activity in specific brain regions and frequency bands. Although emotional perception via different modalities shares common neural mechanisms [54], the pattern of changes in neural activity in response to different sensory inputs varies across frequency bands and brain regions. In particular, the influence of audio was greater than that of the visual stimulus, especially in the delta and alpha bands, with the difference being significantly greater than that of any other group. The emotional components in stimuli are influential for the EEG spectral domain and differ in response to positive and negative emotional stimuli [55]. The pleasure conditions lead to a decrease in delta band PSD and an increase in PSD for both theta and alpha bands. In contrast, unpleasure conditions lead to an increase in delta band PSD and a decrease in PSD in both theta and alpha bands.

It can be clearly observed from the changes in PSD that the influence of audio stimuli was greater than that of visual stimuli, especially for delta and alpha bands. The finding that visual stimulus has a greater influence on PSD than auditory stimuli, particularly in the delta and alpha frequency bands, can be attributed to several factors. First, the visual system has a larger and more direct pathway to the brain than the auditory system, which involves more processing and the integration of information from different sources. This direct pathway allows faster and more efficient transmission of visual information, leading to stronger and more immediate effects on brain oscillations. Furthermore, visual stimuli tend to be more salient and attention-grabbing than auditory stimuli as they provide more detailed and complex information about the environment. This may lead to the greater activation of attention and cognitive processing systems, resulting in stronger and more widespread effects on brain oscillations. It is also possible that the differences observed in this study were due to the specific characteristics of the visual and auditory stimuli used. For example, visual stimuli may have been more engaging for or appealing to the participants, leading to stronger effects on PSD. Similarly, auditory stimuli may have been less salient or more difficult to discriminate, resulting in weaker effects on the PSD [56].

Moreover, by incorporating the results from Section 3.3, the relationship between emotion and stimulus modality on PSD has found that there were some differences between single-modal and multimodal emotions. However, the differences in emotions were not the cause of the significant differences in PSD, which resulted from differences in the stimulus modality itself. Therefore, it can be concluded that in multimodal emotional stimulation, it is often the stimulation modality itself that is responsible for changes in the PSD rather than the emotional state itself.

### 3.5. Temporal ERP Analysis 

**Figure 13:**
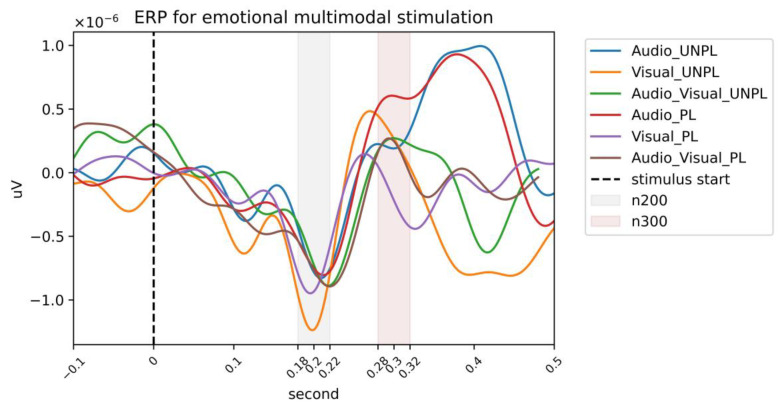
Temporal illustration for six stimulated conditions; those within 180–220 ms are highlighted as N200, those within 280–320 ms are highlighted as P300, and 0 s is the stimulation start time.

To obtain the ERP results, we used the workflow described in the Materials and Methods section. First, we preprocessed the raw data, then selected Cz, C3, C4, P3, Pz, and P4, utilizing the FIR filter to filter them into 1–40 Hz and averaged the data from six channels to obtain the temporal EEG from each condition. We chose the stimulus start time as 0 s, time-locked the time from −500 ms to ~500 ms, and epoched the data. The processed data were used for N200 and P300 calculations, as shown in Figure 13. 

**Figure 14:**
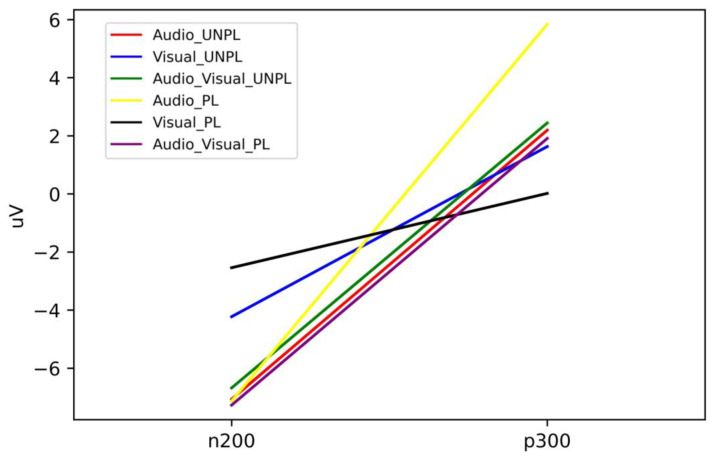
Depiction of potential changes for six stimulated experimental conditions corresponding with N200 and P300 values.

For the calculation of N200, we chose an average potential range of 180–220 ms, and we chose an average potential range of 280–320 ms for P300. Thus, the six conditions for N200 and P300 were obtained as per Table 6 and visualized in Figure 14. 

Here, we investigated the impact of emotional valence and stimulus modality on the amplitude values of the N200 and P300 components of event-related potentials (ERPs). We analyzed the amplitude values of these components under six different conditions, namely, audio unpleasure, visual unpleasure, audio-visual unpleasure, audio pleasure, visual pleasure, and audio-visual pleasure.

Our results showed that the highest amplitude of the N200 component was observed in the visual pleasure condition (−2.54 µV), while the lowest amplitude was found in the audio unpleasure condition (−7.07 µV). Similarly, the highest amplitude of the P300 component was observed in the audio pleasure condition (5.84 µV), while the lowest amplitude was observed in the visual pleasure condition (0.02 µV).

Our findings suggest that the saliency of the emotional cue and the processing efficiency of the sensory modality are important factors in shaping the amplitude values of ERP components. The higher N200 amplitude in the visual pleasure condition may be due to the faster and more efficient processing of visual stimuli compared to auditory stimuli, and the pleasure emotion may have a more salient visual cue [57]. Conversely, the lower N200 amplitude in the audio unpleasure condition may be due to the decreased processing of auditory stimuli in the unpleasure state and reduced attention and concentration [58].

In the case of the P300 component, the higher amplitude value in the audio pleasure condition may be attributed to the more salient auditory cue in the pleasure emotion, which may enhance attention and cognitive processing [59]. The lower amplitude value in the visual pleasure condition may be due to the faster and more efficient processing of visual stimuli, and the pleasure emotion may have a less salient visual cue, leading to a decreased allocation of attentional resources [60].

Therefore, the ERP analysis revealed that the saliency of the emotional cue and the processing efficiency of the sensory modality were important factors in shaping the amplitude values of N200 and P300 components. Emotional information processing in multimodal environments is highly integrated, with interactions and regulatory effects between different sensory channels [20]. The N200 [17] and P300 components were subjected to influences from the emotional multimodal stimulation in the experiment. The highest and lowest amplitudes of the N200 component were observed in visual pleasure and audio unpleasure conditions, respectively. Similarly, the highest and lowest amplitudes of the P300 component were observed in audio pleasure and visual pleasure conditions, respectively. These findings suggest that increased salient sensory cues lead to higher amplitude values for the corresponding ERP component.

## 4. Conclusions

This study investigated the impact of emotional states and stimulus modality on brain activity using three analytical methods: SAM, PSD, and ERP. SAM was used to identify the regions of interest, PSD was used to quantify changes in brain activity in response to different stimuli, and ERP was used to measure the amplitude of N200 and P300 components. Our findings shed light on the neural mechanisms underlying emotional processing across sensory modalities. Highlights: In multimodal emotional stimulation, it is often the stimulation modality itself that is responsible for changes in PSD rather than the emotional state itself. The saliency of emotional cues and the processing efficiency of sensory modality are important factors in shaping the amplitude values of N200 and P300 components. Increased salient sensory cues lead to higher amplitude values for the corresponding ERP components in multimodal emotional environments. Overall, our study provides a comprehensive understanding of the neural mechanisms underlying emotional processing across sensory modalities, mainly via EEG spatiotemporal analysis. Our findings have important implications for understanding the neural mechanisms underlying emotional processing and can aid the development of more effective interventions for emotion-related applications. For future research, we will consider more complex types of multimodal stimuli and their effects on the brain under the role of multimodal emotional stimulation.

## Figures and Tables

**Table 1:** EEG data collected during the experiment.

Audio-Unpleasure	Visual-Unpleasure	Audio-Visual-Unpleasure	Audio-Pleasure	Visual-Pleasure	Audio-Visual-Pleasure	Resting State
200 trails	200 trails	200 trails	200 trails	200 trails	200 trails	20 trails

**Table 2:** Self-assessment results of each subject in different experimental conditions.

	Audio Pleasure	Visual Pleasure	Audio-Visual Pleasure	Audio Unpleasure	Visual Unpleasure	Audio-Visual Unpleasure
Sub01	7.8	8.2	7.4	−5.3	−4.6	−8.3
Sub02	5.2	7.4	6.5	−6.7	−8.3	−6.1
Sub03	6.4	5.5	7.9	−5.0	−6.2	−7.9
Sub04	7.5	4.2	7.8	−8.0	−4.3	−6.8
Sub05	6.0	5.2	7.1	−5.4	−5.2	−7.5
Sub06	5.6	8.0	8.5	−7.0	−3.8	−6.4
Sub07	6.5	7.1	3.8	−6.6	−4.1	−6.7
Sub08	5.2	5.1	6.1	−7.9	−6.7	−8.5
Sub09	7.9	7.7	7.7	−8.5	−5.6	−6.9
Sub10	3.4	5.3	8.2	−5.9	−6.3	−5.1
Sub11	7.2	8.3	7.8	−4.4	−3.6	−7.9
Sub12	7.2	7.4	7.5	−2.7	−7.3	−6.3
Sub13	4.8	6.0	6.0	−8.3	−7.2	−7.0
Sub14	7.6	3.9	8.2	−6.2	−3.3	−6.1
Sub15	6.9	4.7	6.6	−4.8	−8.6	−7.1
Sub16	6.9	7.7	7.0	−4.6	−8.1	−6.3
Sub17	4.7	4.7	8.0	−6.6	−7.0	−7.8
Sub18	6.6	5.2	7.4	−6.2	−5.2	−7.9
Sub19	8.3	7.4	8.0	−8.4	−6.3	−7.5
Sub20	8.2	3.4	7.0	−4.8	−6.4	−5.8
**Average**	**6.50 ± 2.40**	**6.17 ± 2.51**	**7.23 ± 1.91**	**−6.17 ± 2.35**	**−5.90 ± 2.30**	**−7.00 ± 2.02**

**Table 3:** Percentage change in PSD with pre-stimulated/stimulated conditions, considering frequency bands and functional areas. *p*-values are provided in parenthesis.

Stimulus Types	Band	Change in Percentage (%)				
		Frontal	Temporal	Central	Parietal	Occipital
Audio Unpleasure	Delta					
	Theta					
	Alpha					2.177 * (0.0745)
Visual Unpleasure	Delta					
	Theta					
	Alpha		−1.814 ** (0.0434)		−2.449 ** (0.039)	−2.845 ** (0.020)
Audio-visual Unpleasure	Delta					
	Theta					
	Alpha		−1.514 ** (0.0418)		−1.867 ** (0.0412)	−2.176 ** (0.0218)
Audio Pleasure	Delta					
	Theta					
	Alpha		1.465 * (0.0818)			2.696 ** (0.0374)
Visual Pleasure	Delta					
	Theta					
	Alpha		−1.347 * (0.0506)		−1.759 ** (0.0470)	−1.876 ** (0.0382)
Audio-visual Pleasure	Delta					
	Theta					
	Alpha		0.359 ** (0.0236)	0.727 * (0.0740)	0.473 ** (0.0127)	0.070 ** (0.0113)

0.05 < *p* < 0.1 as *, 0.01 < *p* < 0.05 as **.

**Table 4:** Change in PSD with emotional status, considering frequency bands and functional areas.

Stimulus Types	Band	Change in Percentage (%)				
		Frontal	Temporal	Central	Parietal	Occipital
Audio PL-UNPL	Delta			−0.171 ** (0.0221)		
	Theta		0.148 * (0.098)	0.174 ** (0.046)	0.221 ** (0.026)	−0.234 ** (0.0477)
	Alpha		0.411 *** (0.008)		0.362 ** (0.024)	0.699 *** (0.0016)
Visual PL-UNPL	Delta		−0.415 ** (0.011)		−0.253 * (0.0796)	
	Theta		0.134 * (0.0956)			0.238 ** (0.0596)
	Alpha				0.269 ** (0.0378)	0.104 ** (0.0240)
Audio-visual PL-UNPL	Delta	−0.4942 ** (0.0449)		−0.443 *** (0.0032)	−0.4889 *** (0.027)	−0.432 ** (0.011)
	Theta		0.123 ** (0.0132)		0.104 ** (0.0163)	
	Alpha		0.0492 ** (0.0331)	0.031 *** (0.0014)	0.034 ** (0.041)	0.130 *** (0.0018)

0.05 < *p* < 0.1 as *, 0.01< *p* < 0.05 as **, and 0.01 > *p* as ***.

**Table 5:** Changes in PSD with respect to multi-stimulated/uni-stimulated and pleasure/unpleasure conditions, considering frequency bands and functional areas.

Stimulus Types	Band	Change in Percentage (%)				
		Frontal	Temporal	Central	Parietal	Occipital
AV-A UNPL	Delta	−1.205 *** (0.010)	−1.055 *** (0.009)	−1.022 *** (0.002)	−1.014 *** (0.002)	−0.813 *** (0.008)
	Theta	0.607 * (0.063)			0.369 ** (0.040)	0.758 *** (0.003)
	Alpha	0.309 * (0.095)	−2.752 *** (0.0001)	−1.821 *** (0.0001)	−3.382 ** (0.0001)	−4.441 *** (0.0002)
AV-V UNPL	Delta		0.693 *** (0.001)	0.633 *** (0.003)	0.559 *** (0.008)	0.605 *** (0.004)
	Theta	0.838 *** (0.0007)	0.369 *** (0.0007)	0.380 *** (0.0026)	0.362 *** (0.002)	0.300 ** (0.0027)
	Alpha	0.234 ** (0.020)	−0.141 ** (0.030)	−0.141 * (0.075)	−0.182 ** (0.045)	−0.198 ** (0.040)
AV-A PL	Delta	−1.528 ** (0.020)	−0.721 * (0.082)	−1.309 *** (0.004)	−1.381 *** (0.003)	−1.088 ** (0.011)
	Theta		0.123 ** (0.0132)			0.768 ** (0.030)
	Alpha		−3.1379 *** (0.0001)	−1.942 *** (0.0004)	−3.811 ** (0.0002)	−5.060 *** (0.0001)
AV-V PL	Delta	0.525 * (0.095)	0.408 * (0.054)	0.306 * (0.095)	0.335 * (0.080)	0.405 ** (0.043)
	Theta		0.359 *** (0.0017)	0.306 ** (0.014)	0.311 *** (0.004)	0.313 *** (0.005)
	Alpha	0.197 * (0.077)				

0.05 < *p* < 0.1 as *, 0.01< *p* < 0.05 as **, and 0.01 > *p* as ***.

**Table 6:** N200 and P300 values for the six stimulated experimental conditions.

	N200 (μV)	P300 (μV)
Audio_UNPL	−7.07	2.19
Visual_UNPL	−4.23	1.63
Audio-Visual_UNPL	−6.68	2.44
Audio_PL	−7.13	5.84
Visual_PL	−2.54	0.02
Audio-Visual_PL	−7.27	1.91

## Data Availability

Data recorded from our experimental work are currently under further analysis; the data will be uploaded when this process is complete.

## Supplementary Materials

The following supporting information can be downloaded at: https://www.mdpi.com/article/10.3390/s23104801/s1, Supplementary Table S1: The corresponding relationship between experimental videos and sources from the film database (Unpleasure (sad: bs), pleasure: yy), S2. Pseudo-random protocol for the experiment, S3. Questionnaire before emotion EEG experiment.
