# Supplementary material for: Influence of Multimodal Emotional Stimulations on Brain Activity: An Electroencephalographic Study
Source: Sensors (Basel). 2023 May 16;23(10):4801. doi: 10.3390/s23104801 (PMC10221168; doi:10.3390/s23104801)
Supplement: Supplementary file 1 [file sensors-23-04801-s001.zip › sensors-2384403-SI.pdf]

Supplementary Material

Table S1. The corresponding relationship between experimental videos and sources from the film database (Unpleasure (sad: bs), pleasure: yy).

Unpleasure(sad) videos	1	2	3	4	5	6	7	8	9	10
Source from film database	bs1 0:00~0:30	bs2 0:00~0:30	bs6 0:00~0:30	bs11 0:00~0:30	bs12 0:00~0:30	bs13 0:00~0:30	bs15 0:00~0:30	bs16 0:00~0:30	bs1 0:40~1:10	bs2 0:50~1:20
Pleasure videos	1	2	3	4	5	6	7	8	9	10
Source from film database	yy1 0:00~0:30	yy2 0:00~0:30	yy5 0:00~0:30	yy11 0:00~0:30	yy13 0:00~0:30	yy14 0:00~0:30	yy15 0:00~0:30	yy16 0:00~0:30	yy1 0:40~1:10	yy2 0:50~1:20

S2. Pseudo-random protocol for the experiment

Audio-unpleasure stimuli are named 1-10, visual-unpleasure stimuli are named 11-20, and audio–visual are named 21-30 (corresponding to the order of the Supplementary Table).

Audio-pleasure stimuli are named 31-40, visual-pleasure stimuli are named 41-50, and audio-pleasure are named 51-60 (with the order of supplementary table).

Additionally, by using the following Python code, the order of 60 stimuli would be determined for each subject with respect to a pseudorandom order.

```
import random
# Generate 60 pseudorandom integers between 1 and 60
for i in range(60):
    rand_num = random.randint(1, 60)
    print(rand_num)
```

S3. Questionnaire before emotion EEG experiment

Questionnaire time: year month day hour minute

1. Are you left-handed? (Y/N)
2. Did you sleep well last night? (Y/N)
3. Did you have mental illnesses recently, history of mental illness or been treated by a psychotherapist? (Y/N, if YES, please talk to the experimenter immediately)
4. Did you have physical injuries recently? (Y/N, if YES, please talk to the experimenter immediately)
5. Has there been anything that has made you feel seriously emotionally affected recently? (Y/N, if YES, please talk to the experimenter immediately)
6. Do you have hearing or visual impairment (Y/N, if YES, please talk to the experimenter immediately)
